# Spontaneous Hind Limb Paralysis Due to Acute Precursor B Cell Leukemia in RAG1-deficient Mice

**DOI:** 10.1007/s12031-022-02025-7

**Published:** 2022-05-18

**Authors:** Liu Feifei, Anna Richter, Jens Runge, Jonas Keiler, Andreas Hermann, Markus Kipp, Sarah Joost

**Affiliations:** 1 Institute of Anatomy, University Medical Center Rostock, Gertrudenstr 9, 18057 Rostock, Germany; 2 Department of Medicine, Clinic III, Hematology, Oncology, Palliative Medicine, Rostock University Medical Center, Ernst-Heydemann-Straße 6, 18057 Rostock, Germany; 3 Department of Neurology, Translational Neurodegeneration Section “Albrecht-Kossel”, Rostock University Medical Center, Gehlsheimer Str. 20, 18057 Rostock, Germany; 4 Deutsches Zentrum Für Neurodegenerative Erkrankungen (DZNE) Rostock/Greifswald, 18147 Rostock, Germany; 5 Center for Transdisciplinary Neurosciences Rostock (CTNR), Rostock University Medical Center, University of Rostock, 18147 Rostock, Germany

**Keywords:** RAG1-deficient mice, Paralysis, Hematologic Malignancy, Pro B cell leukemia, Micro-CT

## Abstract

RAG1-deficient mice are a frequently used immunodeficient mouse strain lacking mature lymphocytes. Apart from an elevated risk for infections, no predispositions for diseases of this strain have been described so far. We here report a high incidence of spontaneous pro B cell leukemia resulting in hind limb paralysis in our colony of RAG1-deficient mice. At an age of 7–13 months, animals developed hind limb paralysis and rapid decrease of the overall health condition leading to the need of euthanasia. Histological and flow cytometric analyses as well as micro-computed tomography (micro-CT) scans revealed CD45^+^ CD19^+^ IgM^−^ cell infiltrates in the spleen, the bone marrow, and the spinal canal. Monthly blood sampling and screening for CD19^+^ blast frequency in the peripheral blood was successfully established for monitoring of leukemia development before symptom onset. We conclude that facilities that breed RAG1-deficient mice should be aware of the risk of leukemia development in this strain and recommend to implement regular blood sampling for aged RAG1-deficient animals.

**Supplementary Information:**

The online version contains supplementary material available at 10.1007/s12031-022-02025-7.

## Introduction

RAG (recombination activating gene) proteins are necessary for somatic (VDJ) recombination in B and T cell receptor formation and therefore the maturation of all lymphocytes (Lescale and Deriano 2016). Knockdown of RAG1 or RAG2 in mice results in lymphopenia and, therefore, severe combined immunodeficiency (SCID) (Mombaerts et al. 1992; Shinkai 1992). RAG1-deficient mice are used to evaluate the influence of lymphocytes in various experimental models of infections, autoimmunity, tumorigenesis, and metastasis and are therefore a frequently bred immunodeficient mouse strain (Belizario 2009). Like all immunodeficient mice, RAG1-deficient mice should be housed under specific pathogen-free conditions with high hygiene barrier standards. Besides a general elevated susceptibility for infections due to the lack of mature lymphocytes, no predispositions towards specific diseases in RAG1-deficient mice have been reported yet.

In our facility, a colony of RAG1-deficient mice was used to examine if a drug-induced effect on the central nervous system was mediated by lymphocytes or a different cell type. During breeding of RAG1-deficient mice for this project, we observed a high incidence of spontaneous hind limb paralysis with a sudden and rapid decrease of the general health condition in animals aged 7 to 14 months, leading to the need of euthanasia. Analysis of spleens, spines, bone marrow, and peripheral blood revealed pro B cell leukemia as the probable cause for these symptoms. As a consequence, monthly monitoring of CD19^+^ cells in the peripheral blood was used to diagnose leukemia development before onset of symptoms in our colony.

## Methods

### Animals

RAG1-deficient mice were originally purchased from Jackson Laboratories (B6.129S7-Rag1^tm1Mom^/J, #002216) and then bred in the animal facility of the Department of Neurology with Institute of Translational Neurology (University of Münster, Münster, Germany). Fifteen RAG1-deficient mice (8 female, 7 male) were kindly provided by Sven Meuth (formerly Department of Neurology with Institute of Translational Neurology, University of Münster, Münster, Germany) and then housed in the animal facility of the Institute of Anatomy Rostock under specific pathogen-free conditions in a positive-pressured isolator unit under a 12 h light-dark-cycle with standard chow *ad libitum*. This cohort of mice (hereinafter called generation *x*) and one generation of their offspring (10 males, 7 females, generation *x* + 1) was observed for this report. For wildtype control tissue, age-matched C57BL6/J animals were provided by the accredited laboratory animal Core Facility of the Rostock University Medical Center.

Annual testing of the hygiene status in the animal room was performed following the Federation-of-European-Laboratory-Animal-Science-Associations (FELASA) recommendations in sentinel animals confronted with embedding from all cages of the respective room (complete list in Supplemental material 1). This testing showed no detectable pathogens. All procedures involving animals were conducted according to the FELASA recommendations and in agreement with the local authorities (Landesamt für Landwirtschaft, Lebensmittelsicherheit und Fischerei Mecklenburg-Vorpommern).

### Transcardial Perfusion and Immunohistochemistry

Mice of generation *x* showing complete or partial hind limb paralysis were immediately (within 6 h after symptom observation) anesthetized with ketamine/xylazine, euthanized, and transcardially perfused with 20 ml of phosphate-buffered saline (PBS) and 40 ml of 3.7% formaldehyde in PBS. Spleens, lymph nodes, and spines (after decalcification in 10% EDTA solution for 2 weeks) were embedded in paraffin for histological and immunohistochemical analyses.

For immunohistochemistry, sections were deparaffinized, unmasked by heat-induced antigen retrieval in citrate (pH 6) or Tris/EDTA (pH 9) buffer; intrinsic peroxidase activity was blocked with 0.35% H_2_O_2_; and unspecific bindings were blocked with 5% normal goat serum (Vector Laboratories, S1000) in PBS for 1 h at room temperature. Sections were incubated with primary antibodies (CD44, 1:1000, RRID:AB_2885107; CD45, 1:100, RRID:AB_442810; CD45R, 1:400, RRID:AB_467254; CD34, 1:500, RRID:AB_306316; CD3, 1:1000, RRID:AB_443425; CD19, 1:1000, AB_2895109; CD20, 1:100, RRID:AB_1139386; Ki67, 1:10,000, RRID:AB_443209) overnight at 4 °C and were incubated with secondary antibodies (1:200, goat-anti-rabbit RRID:AB_2313606, goat-anti-rat RRID:AB_2336202) for 2 h at room temperature. Staining was visualized by Elite ABC-Kit (Vectastain) and DAB (3,3′-diaminobenzidine, Dako K3468) or EnVision + (Dako K400311-2) following the manufacturer’s instructions (Kaddatz et al. 2021). Low-intensity hematoxylin counterstaining was performed to identify the cell nuclei and to ease the overall orientation within the section. For IgM staining, sections were incubated in AlexaFluor 488-coupled anti-IgM (1:200, RRID:AB_2801490) and counterstained with DAPI. All stains were performed on wild-type tissue in parallel, and appropriate positive and negative controls were performed to ensure antibody specificity (data not shown). Stained sections were documented using a brightfield/epifluorescence microscope (Leica DM6 B) and the software Leica Application Suite X (version 3.7.0.20979).

### Flow Cytometric Analysis

Mice of generation *x* + 1 with signs of emerging hind limb paralysis or elevated CD19^+^ blast levels in the peripheral blood were anesthetized with ketamine/xylazine, and the animal was exsanguinated via the retrobulbar venous plexus. After cervical dislocation for confirmation of death, spleen and femoral bones were dissected and collected in cold PBS. Bone marrow and spleen cells were isolated as described before (Richter et al. 2020). For flow cytometric characterization of the blast population using FACSVerse (Beckton Dickinson) and FACSuite software, the following antibodies were used: CD3e-FITC, CD11b-FITC, CD8a-PE (all Miltenyi Biotec), CD34-FITC, CD45R-FITC, CD3e-PE, Sca-1-PE, CD4-APC, c-kit-APC, CD45-PerCP-Cy5.5 (all BD), CD19-PE, IgM-APC (all Biolegend). Doubling times of leukemic cell populations were calculated from peripheral blood blast frequencies using the following formula with *t*_1_ and *t*_2_ indicating the age of the animal in days at the respective time points of sample analysis:

$$\mathrm{Doubling\;time}=\frac{\left({t}_{2}- {t}_{1}\right)\times \mathrm{log}(2)}{\mathrm{log}(\mathrm{blast\;frequency}{(t}_{2}))- \mathrm{log}(\mathrm{blast\;frequency}({t}_{1}))}$$

Cytospins were prepared from spleen and bone marrow cell suspensions and stained as described before (Richter et al. 2019). For monitoring of CD19^+^ blast frequencies, blood (< 50 µl) was sampled from the tail vein, and CD19^+^, c-kit^+^ and Sca-1^+^ cell populations were quantified as described above.

### Micro-CT

Two wild-type and two RAG1-deficient animals were transcardially perfused directly after the onset of hind limb paralysis as described above, and the spine was dissected. Spines were incubated in 1% Lugol’s iodine (aqueous iodine-potassium iodide) for at least 7 days for soft tissue contrast enhancement. For micro-CT scans, samples were mounted in tubes filled with PBS. Virtual image stacks were obtained by micro-CT scans of the lumbar vertebrae using a Phoenix Nanotom 180 (Phoenix|X-ray, GE Sensing & Inspection Technologies) in high-resolution mode (target: molybdenum, mode: 0). Analysis of micro-CT data and the visualization was conducted with the software Imaris (version 8.4, Bitplane, Switzerland). Automated segmentations of the vertebrae were applied by adjusting the contrast values manually. Segmentations were used for final masking and to prepare the images and 3D-reconstructions.

## Results

Of the 15 RAG1-deficient mice of generation *x*, 10 mice developed complete or partial hind limb paralysis at an age between 7 and 13 months and were euthanized. Three mice were euthanized due to sudden but massive decrease of general condition (apathy, abnormal posture, at 9 and 12 months of age) and two mice died spontaneously without any signs of disease (10 and 12 months of age).

**Fig. 1:**
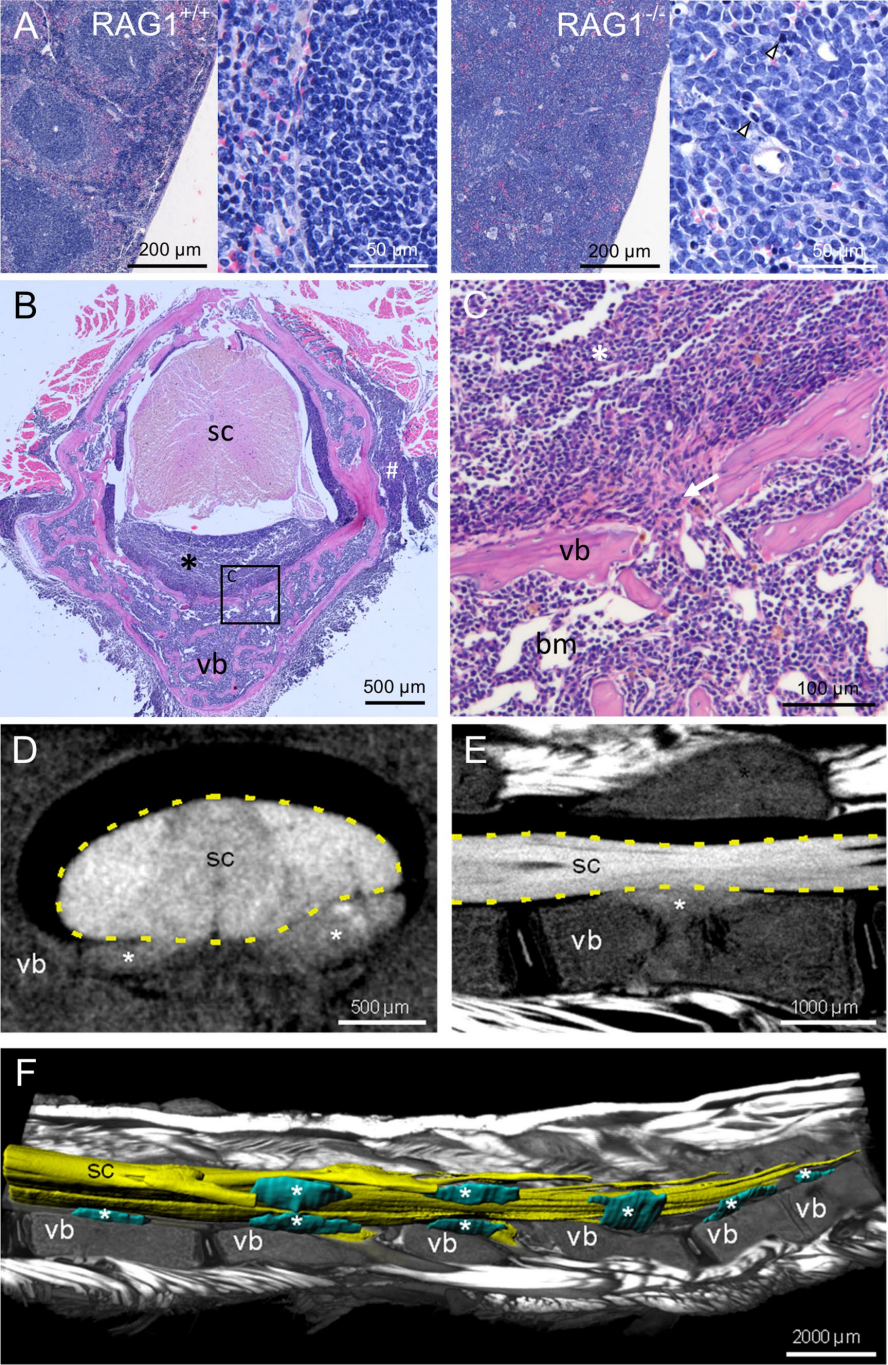
Morphological changes in spine and spleen of paralysis-affected RAG1-deficient mice. **A** Hematoxylin–eosin staining of spleen (paraffin) sections of wild-type controls and RAG1-deficient mice with hind limb paralysis (arrowheads highlight mitotic figures). **B** Hematoxylin–eosin staining of decalcified spine sections from RAG1-deficient mice with hind limb paralysis reveals cellular infiltrates (asterisk) in the spinal canal between vertebral bone (vb) and spinal cord (sc). **C** Enlarged view of B (square) showing discontinuities (arrow) of the vertebral bone (vb), connecting bone marrow (bm) and infiltrates (asterisk) in the spinal canal. **D** Virtual cross section and **E** parasagittal section through the spinal cord (sc) and vertebral bone (vb) based on micro-CT showing the cellular infiltrates (asterisks) in the spinal canal. **F** Ventrolateral view of the lumbar spine with parasagittal section through the vertebrae and muscles (volume rendering) to expose the 3D-reconstructed (surface rendering) spinal cord (sc) and cellular infiltrates (asterisks)

Eight RAG1-deficient mice with paralysis were dissected after euthanasia. Spleens were significantly enlarged. Lymph nodes differed in size ranging from normal to greatly enlarged. A small solid tissue mass of approximately 3 mm diameter was found located dorsally to the left scapula of one mouse. No other macroscopic abnormalities were found. Spines, spleens, lymph nodes, and the tissue mass were processed for histological evaluations. Hematoxylin–eosin staining of spleens and lymph nodes demonstrated loss of the follicular structure of wild-type lymphoid organs, and instead, high numbers of uniformly shaped medium sized cells with large pale nuclei were found (Fig. 1A). Occasionally, mitotic figures were observed in these cells (arrowheads in Fig. 1A). The solid tissue mass dissected from one animal as described above showed a very similar cellular morphology (data not shown).

In spine sections, the bone marrow cavities of vertebral bones were filled with tightly packed uniform cells of comparable morphology (medium-sized cells with large pale nuclei) instead of the well-structured bone marrow cytoarchitecture in wild-type mice (Fig. 1B, C, vertebral bone marked as vb). In all analyzed animals, cell infiltrates were found in the spinal canal between the vertebral bone and the meninges surrounding the spinal cord (asterisk in Fig. 1B, C). Occasionally, cell-filled openings in the vertebral bone connecting the bone marrow cavity with the spinal canal were observed (highlighted by arrow in Fig. 1C). The spinal cord tissue did not show any abnormalities. On the dorsal and lateral sides of the vertebrae, the described cells also infiltrated into back muscles (marked by # in Fig. 1B).

To assess the extent of cellular infiltrates along the whole spinal canal of paralysis-affected RAG1-deficient mice, we performed micro-CT scanning of the spine of two affected animals after post-fixation immersion contrasting. Multiple infiltrates were found in the lumbar spinal canal in close proximity to the ventral and dorsal roots of the spinal cord. Virtual cross-sections showed that infiltrates mainly spread on the ventral side of the spinal cord, oriented towards the vertebral bone bodies and tended to compress the spinal cord (Fig. 1D, infiltrates marked by asterisks). Parasagittal sectioning revealed that infiltrates were positioned at the center of vertebral bones and, confirming the histological findings, pervading the vertebral bone towards the bone marrow cavity (Fig. 1E). For complete assessment of the spine’s anatomy, cellular infiltrates, the spinal cord, vertebral bones, and surrounding muscles were reconstructed three-dimensionally, revealing that infiltrates occurred along six vertebral bones in the presented animal (Fig. 1F, infiltrates labeled by asterisk).

**Fig. 2:**
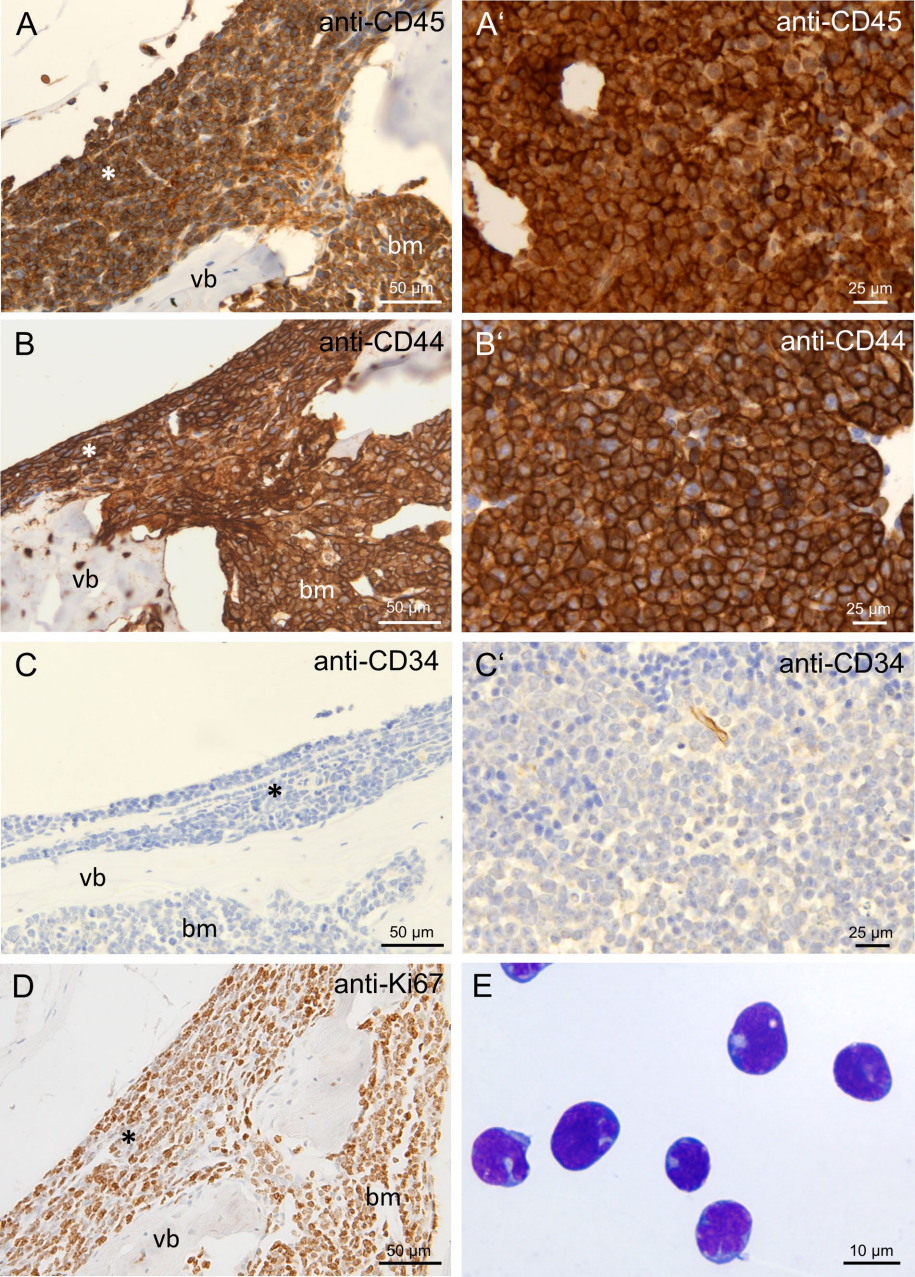
Immunohistochemical evaluation of hematopoietic markers in cellular infiltrates in paralysis-affected RAG1-deficient mice. Immunohistochemical stainings of decalcified spine sections with cellular infiltrates in the spinal canal (asterisk) and the bone marrow cavity (bm) of vertebral bones (vb) and spleen of RAG1-deficient mice with hind limb paralysis. Anti-CD45 staining in spine **A** and spleen **A′**, anti-CD44 staining in spine **B** and spleen **B′**, anti-CD34 staining in spine **C** and spleen **C′**, anti-Ki67 staining in spine **D**, Pappenheim staining of a cytospin preparation of spleen cells with lymphocytic blast morphology **E**

**Fig. 3:**
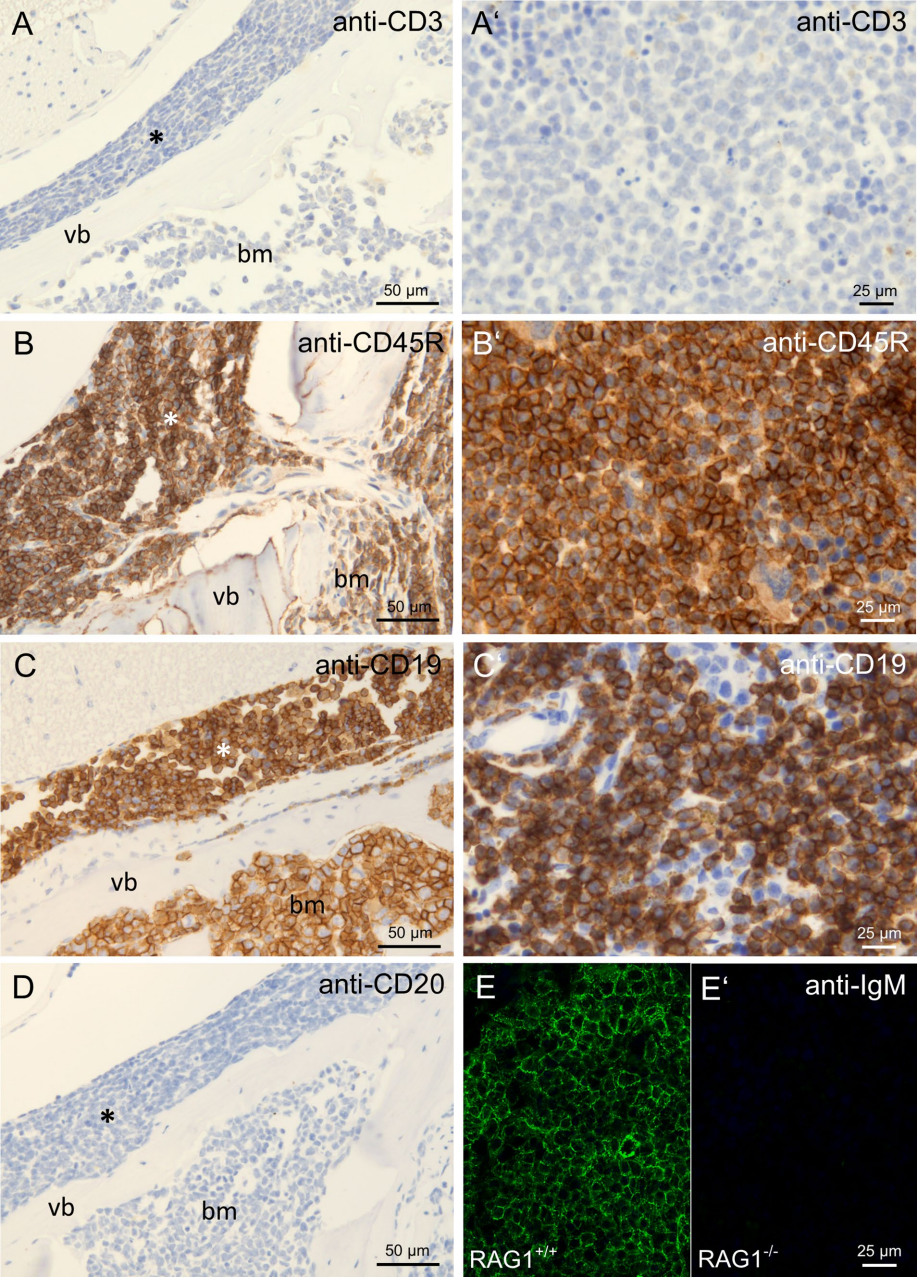
Immunohistochemical evaluation of lymphoid markers in cellular infiltrates in paralysis-affected RAG1-deficient mice. Immunohistochemical stainings of spine with cellular infiltrates in the spinal canal (asterisk) and the bone marrow cavity (bm) of vertebral bones (vb) and spleen of RAG1-deficient animals with hind limb paralysis. Anti-CD3 staining in spine **A** and spleen **A′**, anti-CD45R staining in spine **B** and spleen **B′**, anti-CD19 staining in spine **C** and spleen **C′**, anti-CD20 staining in spine **D,** anti-sIgM staining in wildtype animals **E** and paralysis-affected RAG1-deficient animals **E′**

Due to cell morphology and localization in bone marrow and lymphatic organs, we hypothesized that the observed cells consist of hematopoietic malignant leukemia or lymphoma cells. For further characterization, we performed immunohistochemistry on spleen and bone marrow cells of RAG1-deficient animals with hind limb paralysis. All infiltrating cells were positively stained for CD45 (Fig. 2A), a marker of all hematopoietic cells, as well as CD44 (Fig. 2B), a marker for hematopoietic stem and progenitor cells. However, the cells did not stain positive for CD34 (Fig. 2C), another marker of hematopoietic stem and progenitor cells. The proliferation marker Ki67 stained positive on a high ratio of infiltrating cells (Fig. 2D). Pappenheim-stained cytospin preparations of bone marrow and spleen cells demonstrated cells of typical lymphocytic blast (immature or precursor lymphocytic cell) morphology (Fig. 2E). Subsequently, we tested if the infiltrating cells belonged to the T or B lymphocyte lineage. No staining was observed for CD3 (Fig. 3A), a marker for T lymphocytes. The cells stained positive for CD45R (B220, Fig. 3B), a B lymphocyte marker expressed from the pre-pro-B stage until maturation and CD19 (Fig. 3C), a marker expressed on B lymphocytes from the pro-B cell stage until maturation. Staining for CD20, expressed on B lymphocytes beginning at the pre-B cell stage, was negative (Fig. 3D). Staining for surface-bound IgM (sIgM), expressed by B lymphocytes beginning at the stage of immature B cells, was negative (Fig. 3E).

**Fig. 4:**
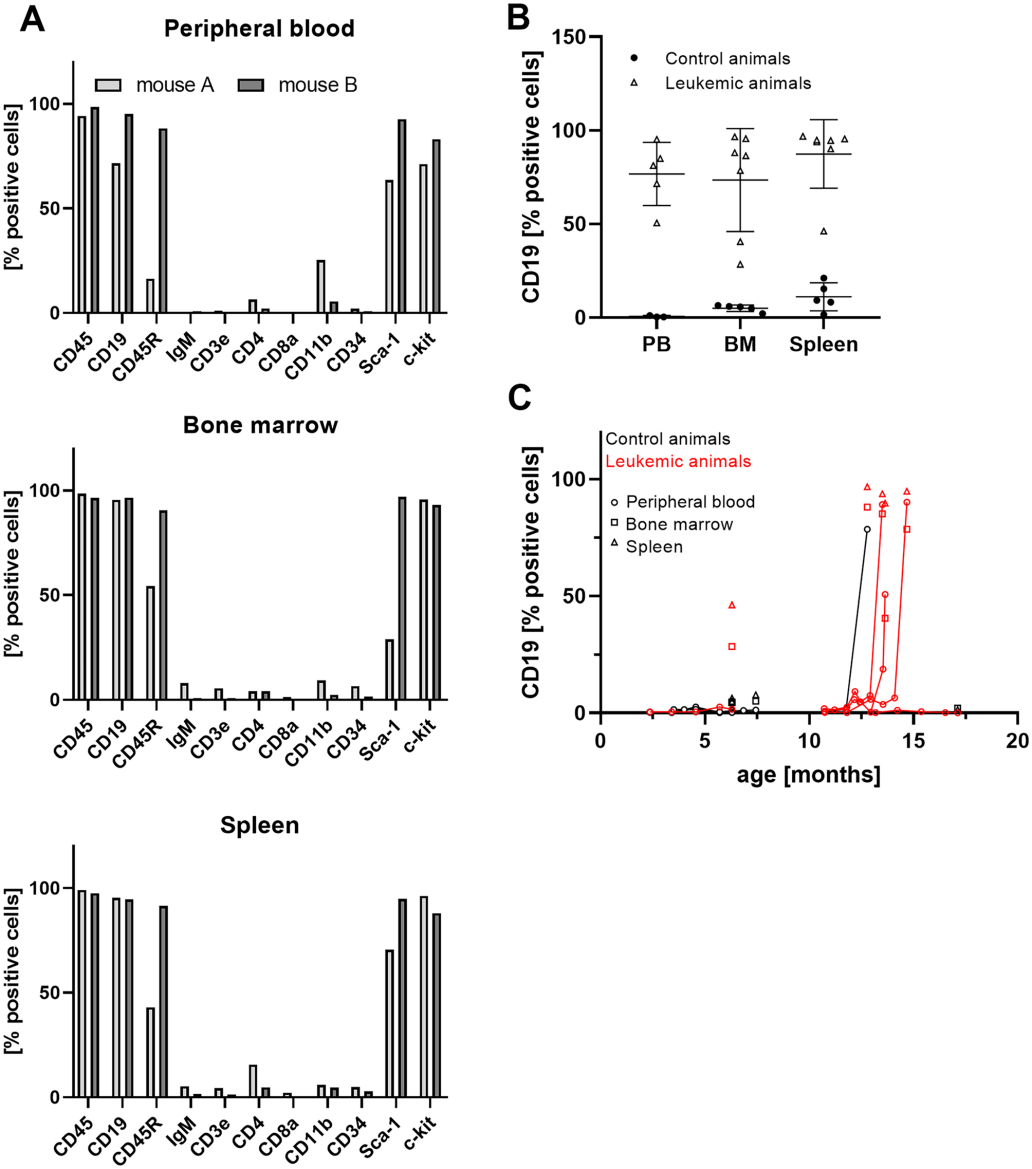
Flow cytometric analysis of spleen, bone marrow, and peripheral blood in paralysis-affected RAG1-deficient mice and controls. **A** Characterization of the tumor cell population was carried out in peripheral blood, bone marrow, and spleen cells of two representative paralyzed animals. **B** Frequency of CD19^+^ cells in peripheral blood (PB), bone marrow (BM), and spleens of leukemic (open triangles) and control animals (filled circles). Mean ± SD. **C** Time-dependent development of the CD19^+^ tumor cell population in affected animals (red) while no leukemic blasts were detected in designated controls (black). Tumor cell frequencies in blood, bone marrow and spleens are displayed as circles, squares, and triangles, respectively. All graphs show the ratio of the concerning cell type in relation to all live cells

Meanwhile, animals of generation *x* + 1 reached the age of susceptibility, and 2 out of 17 animals developed spontaneous hind limb paralysis at an age of 9 months. For confirmation and extension of the immunohistochemical characterization, we performed flow cytometric analyses of peripheral blood cells, bone marrow cells, and isolated splenocyte suspensions of these animals. This analysis confirmed the results of immunohistochemistry, strongly arguing for a precursor B-cell-derived hematologic malignancy. All analyzed tissues were dominated by a population of CD45^+^ CD19^+^ IgM^−^ cells (Fig. 4A). Furthermore, this population stained partly positive for the stem cell and tumor markers c-kit and Sca-1. Differences in the amount of CD45R- and Sca1-positive cells between the two analyzed animals hinted towards slight individual differences of the developmental stage of the dominating cell population. Lack of surface IgM expression further ruled out the possibility of a mature B-cell neoplasia or lymphoma. No considerable numbers of CD3^+^, CD4^+^, or CD8^+^ T lymphocytes or CD11b^+^ macrophages were found. In contrast to the leukemic animals, controls did not exhibit relevant numbers of CD19^+^ blasts in blood, bone marrow, or spleen (Fig. 4B).

To detect early leukemia development in the remaining animals of generation *x* + 1 before onset of leukemia-associated symptoms, we then started to perform monthly screenings for elevated CD19^+^ blast populations in the peripheral blood of all RAG1-deficient animals in our colony (Fig. 4C). If the amount of these cells exceeded 1%, the screening frequency was raised. At a CD19^+^ fraction of more than 10%, the animals were sacrificed before they could develop hind limb paralysis or general decrease of health condition. We identified six more mice (out of 15 animals) with leukemia in the developing stage before onset of symptoms at ages from 11 to 14 months. The CD19^+^ fraction expanded rapidly and displayed mean doubling times of 4.3 days (ranging from 2.1 to 5.5 days) once stable blast proliferation was detected. After diagnosis, mice were euthanized before they could experience severe symptoms of leukemia in accordance with animal welfare regulations.

## Discussion

From the immunohistochemical and flow cytometric characterization of infiltrating cells in the spinal canal and lymphatic organs, we conclude that the animals developed leukemia of the B cell lineage at the stage of pro B cells. Neoplastic cells from the bone marrow of vertebral bones invaded into the spinal canal, probably by the recently described mechanism of migration along the extracellular laminin sheath of basivertebral veins through osseous canals connecting the bone marrow and spinal canal (Yao et al. 2018). The resulting cellular infiltrates in the spinal canal presumably caused mechanic compression of the spinal cord as well as dorsal and ventral roots, leading to movement disorders like hind limb paralysis. It is hard to judge from our sections if neoplastic cells also penetrate the Dura mater and reach the subarachnoid space at least in some of the analyzed animals. Infiltration of the subarachnoid space by leukemic cells is referred to as Meningeosis leucaemica (a subform of Meningeosis neoplastica (Djukic et al. 2017)) and is not infrequent in human leukemia patients (5–15% of patients with leukemia and lymphoma are affected (Chamberlain 2008)). The clinical presentation is multifaceted with cerebral, cranial nerve, and spinal neurologic symptoms, among them lower motor weakness (Grossman and Krabak 1999). This could be an additional explanation for hind limb paralysis in RAG-deficient animals besides mechanical compression especially of ventral roots.

The incidence of leukemia was remarkably high in our mouse colony (10/15 (67%) animals in generation *x*, 7/17 (41%) animals in generation *x* + 1). It is possible that this high susceptibility for leukemia is unique to our breeding group due to the establishment of unknown mutations after multiple generations of breeding or an undetected infection with a pathogen not covered by hygiene monitoring based on FELASA recommendations (e.g., murine leukemia virus) which, however, detected no pathogens in our facility. Nonetheless, neoplastic changes, especially of hematologic origin, have been described in association with primary immunodeficiency in mice as well as human patients (Chiu et al. 2002; Huang et al. 2011; Verhoeven et al. 2018). This elevated risk of neoplasia might be based on impaired anti-tumor immune responses caused by lymphopenia. Additionally, protein defects that cause immunodeficiency often play vital roles in DNA repair mechanisms or other mechanisms relevant for tumorigenesis and therefore might promote tumor development (Hauck et al. 2018). Furthermore, expression of catalytically inactive RAG1 has been shown to accelerate progression of chronic lymphocytic leukemia in a leukemia-prone mouse line (Nganga et al. 2013).

We therefore assume that spontaneous development of leukemia in RAG1-deficient mice is also likely in other breeding colonies. Awareness of this circumstance is the prerequisite for avoiding animal suffering by striving for early diagnosis and euthanasia before symptom onset. Furthermore, experimental data obtained from RAG1-deficient mice might be affected by undetected development of leukemia. Monthly sampling of small amounts of peripheral blood (< 50 µl) proved effective in our colony to identify leukemia development in early stages before noticeable animal suffering occurred. We therefore recommend to consider regular blood sampling as soon as RAG1-deficient mice reach ages higher than 6 months.

## Supplementary Information

Below is the link to the electronic supplementary material.

Supplementary file1 (PDF 58 KB)

## Data Availability

The datasets generated during and/or analyzed during the current study are available from the corresponding author on reasonable request.

## References

[CR1] Belizario JE (2009). Immunodeficient Mouse Models: an Overview TOIJ.

[CR2] Chamberlain MC (2008). Neoplastic meningitis. Oncologist.

[CR3] Chiu PPL, Ivakine E, Mortin-Toth S, Danska JS (2002). Susceptibility to lymphoid neoplasia in immunodeficient strains of nonobese diabetic mice. Cancer Res.

[CR4] Djukic M, Trimmel R, Nagel I, Spreer A, Lange P, Stadelmann C, Nau R (2017). Cerebrospinal fluid abnormalities in meningeosis neoplastica: a retrospective 12-year analysis. Fluids Barriers CNS.

[CR5] Grossman SA, Krabak MJ (1999). Leptomeningeal carcinomatosis. Cancer Treat Rev.

[CR6] Hauck F, Voss R, Urban C, Seidel MG (2018). Intrinsic and extrinsic causes of malignancies in patients with primary immunodeficiency disorders. J Allergy Clin Immunol.

[CR7] Huang P, Westmoreland SV, Jain RK, Fukumura D (2011). Spontaneous nonthymic tumors in SCID mice. Comp Med.

[CR8] Kaddatz H, Joost S, Nedelcu J, Chrzanowski U, Schmitz C, Gingele S, Gudi V, Stangel M, Zhan J, Santrau E, Greiner T, Frenz J, Müller-Hilke B, Müller M, Amor S, van der Valk P, Kipp M (2021). Cuprizone-induced demyelination triggers a CD8-pronounced T cell recruitment. Glia.

[CR9] Lescale C, Deriano L (2016) V(D)J Recombination: orchestrating diversity without damage. In: Encyclopedia of Cell Biology. Elsevier, pp 550–566

[CR10] Mombaerts P, Iacomini J, Johnson RS, Herrup K, Tonegawa S, Papaioannou VE (1992). RAG-1-deficient mice have no mature B and T lymphocytes. Cell.

[CR11] Nganga VK, Palmer VL, Naushad H, Kassmeier MD, Anderson DK, Perry GA, Schabla NM, Swanson PC (2013). Accelerated progression of chronic lymphocytic leukemia in Eμ-TCL1 mice expressing catalytically inactive RAG1. Blood.

[CR12] Richter A, Roolf C, Hamed M, Gladbach YS, Sender S, Konkolefski C, Knübel G, Sekora A, Fuellen G, Vollmar B, Murua Escobar H, Junghanss C (2019). Combined casein kinase II inhibition and epigenetic modulation in acute B-lymphoblastic leukemia. BMC Cancer.

[CR13] Richter A, Sender S, Lenz A, Schwarz R, Hinz B, Knuebel G, Sekora A, Murua Escobar H, Junghanss C, Roolf C (2020). Influence of Casein kinase II inhibitor CX-4945 on BCL6-mediated apoptotic signaling in B-ALL in vitro and in vivo. BMC Cancer.

[CR14] Shinkai Y (1992). RAG-2-deficient mice lack mature lymphocytes owing to inability to initiate V(D)J rearrangement. Cell.

[CR15] Verhoeven D, Stoppelenburg AJ, Meyer-Wentrup F, Boes M (2018). Increased risk of hematologic malignancies in primary immunodeficiency disorders: opportunities for immunotherapy. Clin Immunol.

[CR16] Yao H, Price TT, Cantelli G, Ngo B, Warner MJ, Olivere L, Ridge SM, Jablonski EM, Therrien J, Tannheimer S, McCall CM, Chenn A, Sipkins DA (2018). Leukaemia hijacks a neural mechanism to invade the central nervous system. Nature.

