# Supplementary material for: Spontaneous Hind Limb Paralysis Due to Acute Precursor B Cell Leukemia in RAG1-deficient Mice
Source: J Mol Neurosci. 2022 May 18;72(8):1646–55. doi: 10.1007/s12031-022-02025-7 (PMC9374608; doi:10.1007/s12031-022-02025-7)
Supplement: Supplementary file 1 — Supplementary file1 (PDF 58 KB) [file 12031_2022_2025_MOESM1_ESM.pdf]

Spontaneous hind limb paralysis due to acute precursor B cell leukemia in RAG1-deficient mice

Liu Feifei, Anna Richter, Jens Runge, Jonas Keiler, Andreas Hermann, Markus Kipp and Sarah Joost

Journal of Molecular Neuroscience

Corresponding author: Markus Kipp, Institute of Anatomy, University Medical Center Rostock, markus.kipp@med.uni-rostock.de

Supplemental material 1: Pathogens tested for annual health monitoring

Two sentinel mice from each animal room, confronted with used embedding from all other cages of that room for 4 weeks before testing.

Viruses:
MHV (mouse hepatitis virus)
EDIM (epizootic diarrhea of infant mice)
MNV (murine norovirus)
MVM (minute virus of mice)
MPV (mouse parvovirus)
TMEV (Theilers murine encephalomyelitis virus)
LCMV (lymphocytic choriomeningitis virus)
MAdV1 (mouse adenovirus 1)
MAdV2 (mouse adenovirus 2)
ECTV (ectromelia virus)
PVM (pneumonia virus of mice)
Reo (reovirus type 3)

Bacteria, mycoplasma, fungi:
Helicobacter spp.
Rodentibacter spp.
Rodentibacter heylii/pneumotropicus
Streptococci ß-haemolytic (not group D)
Streptococcus pneumoniae
Citrobacter rodentium
Clostridium piliforme (Tyzzer's disease)
Corynebacterium kutscheri
Mycoplasma pulmonis
Salmonella spp.
Streptobacillus moniliformis

Ectoparasites:
Arthropodes

Endoparasites:
Aspiculuris spp.
Syphacia spp.
Chilomastix spp.
Coccidia spp.

Entamoeba spp.
Giardia spp.
Spironucleus muris
Tritrichomonas spp.
Flagellates
